# Giant cell tumor of soft tissue involving thyroid gland: a case report and review of the literature

**DOI:** 10.1186/s13256-024-04450-1

**Published:** 2024-03-22

**Authors:** Jianrong Chen, Haiyong Zhang, Xiufang Li, Mengjun Hu, Xiaomin Dai

**Affiliations:** 1 Department of Pathology, Zhuji People’s Hospital of Zhejiang Province, Jianming Road, Taozhu Street, Zhuji, Zhejiang China; 2 Department of Pathology, Zhejiang Hospital, 1229 Gudun Road, Xihu District, Hangzhou, Zhejiang China

**Keywords:** Thyroid gland, Tumor of soft tissue, Giant cell tumor of soft tissue, Immunohistochemistry, Differential diagnosis

## Abstract

**Background:**

Giant cell tumor of soft tissue is a low malignant uncommon neoplasm, with histologic features and immunophenotype similar to its bone counterpart. Primary giant cell tumor of soft tissue in the thyroid gland is considered an exceedingly rare entity.

**Case presentation:**

We describe a case of primary thyroid giant cell tumor of soft tissue in a 69-year-old Chinese female patient. Neck ultrasonography showed a 19 mm × 12 mm × 5 mm nodule with heterogeneous echo and clear boundary located within the left thyroid. Histopathological examination revealed that the neoplasm was composed of two morphological components, mononuclear cells admixed with multinucleated osteoclast-like giant cells. Immunohistochemically, the tumor cells were positive for CD68 and vimentin, but were negative for epithelial membrane antigen, cytokeratin, and additional muscle markers. She underwent left unilateral thyroidectomy, and total thyroidectomy was performed for local recurrence 3 months later. The patient remained well without recurrence or metastasis following up for 12 months.

**Conclusion:**

The significance of this case lies in its rarity, the challenge of preoperative clinical diagnosis, and the differential diagnosis with other malignancies.

## Introduction

Giant cell tumor of soft tissue (GCT-ST) is a relatively rare hyperplastic neoplasm, with morphological characteristics similar to those arising from the bone [1, 2]. Salm *et al*. first described the presence of GCT-ST in 1972, characterized by microscopically detected mixtures of mononuclear cells and multicellular osteoclast-like giant cells [3]. This tumor has a mostly benign clinical course, but tends to local recurrence with a rate of approximately 15%, and only rarely metastasizes to the lung [4–6]. To the best of our knowledge, cases of thyroid giant cell tumor of bone (GCTB) in the literature are sparse, and GCT-ST involving the thyroid are extremely rare and underrecognized. Here, we present a case of primary thyroid GCT-ST and describe the clinicopathological features and differential diagnosis of this entity.

## Case presentation

**Fig. 1:**
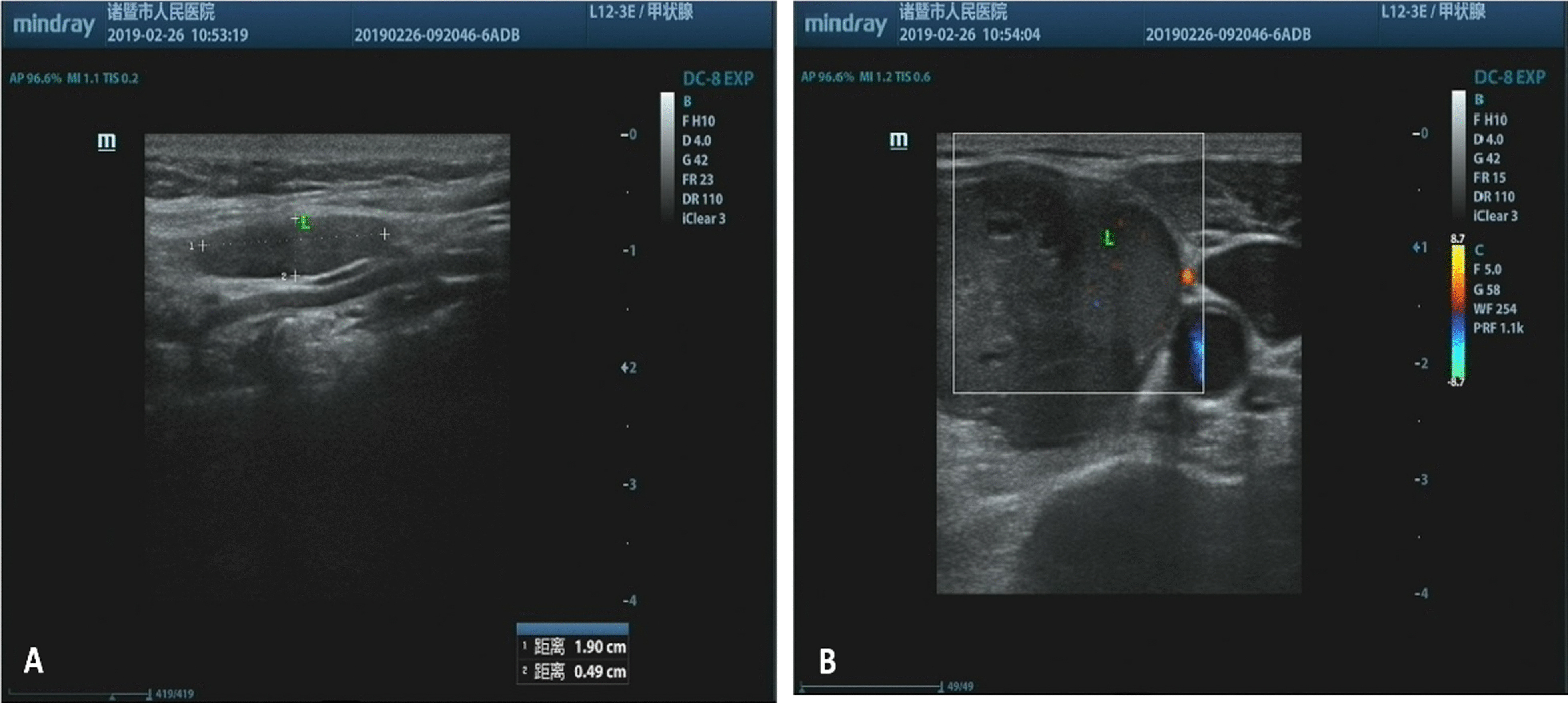
Neck ultrasonography showing an elliptical tumor with soft tissue density and ill-defined boundary adhered to the left lobe of the thyroid (**A** and **B**)

A 69-year-old Chinese female patient presented to our hospital complaining of a recent accidental self-detected mass on the left side of her neck. She had no other obvious symptoms, including local pain, fever, hoarseness, or dysphagia, and also denied personal history of thyroid disease and relevant family history. The palpable, firm, non-tender, and well-defined mass on the left thyroid was enlarged at stage 2 with the ability to move with swallowing during the physical examination. No axillary, supraclavicular, or subclavian lymph node lesion were found. Neck ultrasonography showed a 19 mm × 12 mm × 5 mm nodule with heterogeneous echo and clear boundary located within an enlarged left thyroid lobe (Fig. 1). Laboratory evaluation showed the levels of thyroid stimulating hormone (TSH) normal at 2.66 mIU/L, triiodothyronine (T3) at 1.73 nmol/L, thyroxine (T4) at 104.8 nmol/L, free thyroxine (FT4) at 1.73 nmol/L, free triiodothyronine (FT3) at 104.8 nmol/L, respectively, antithyroid peroxidase antibodies (A-TPO) at 106.1 IUmol/mL, anti-thyroid microsome anti-body (ATMA) at 382.9 IU/mL, and anti-thyroglobulin antibody (TGAB) at 990 IU/mL. Routine blood examination, serum amylase, glucose, and liver and kidney function were all within the normal range. Further abdominal computed tomography (CT) and general chest x-ray revealed no evidence of other abnormalities.

**Fig. 2:**
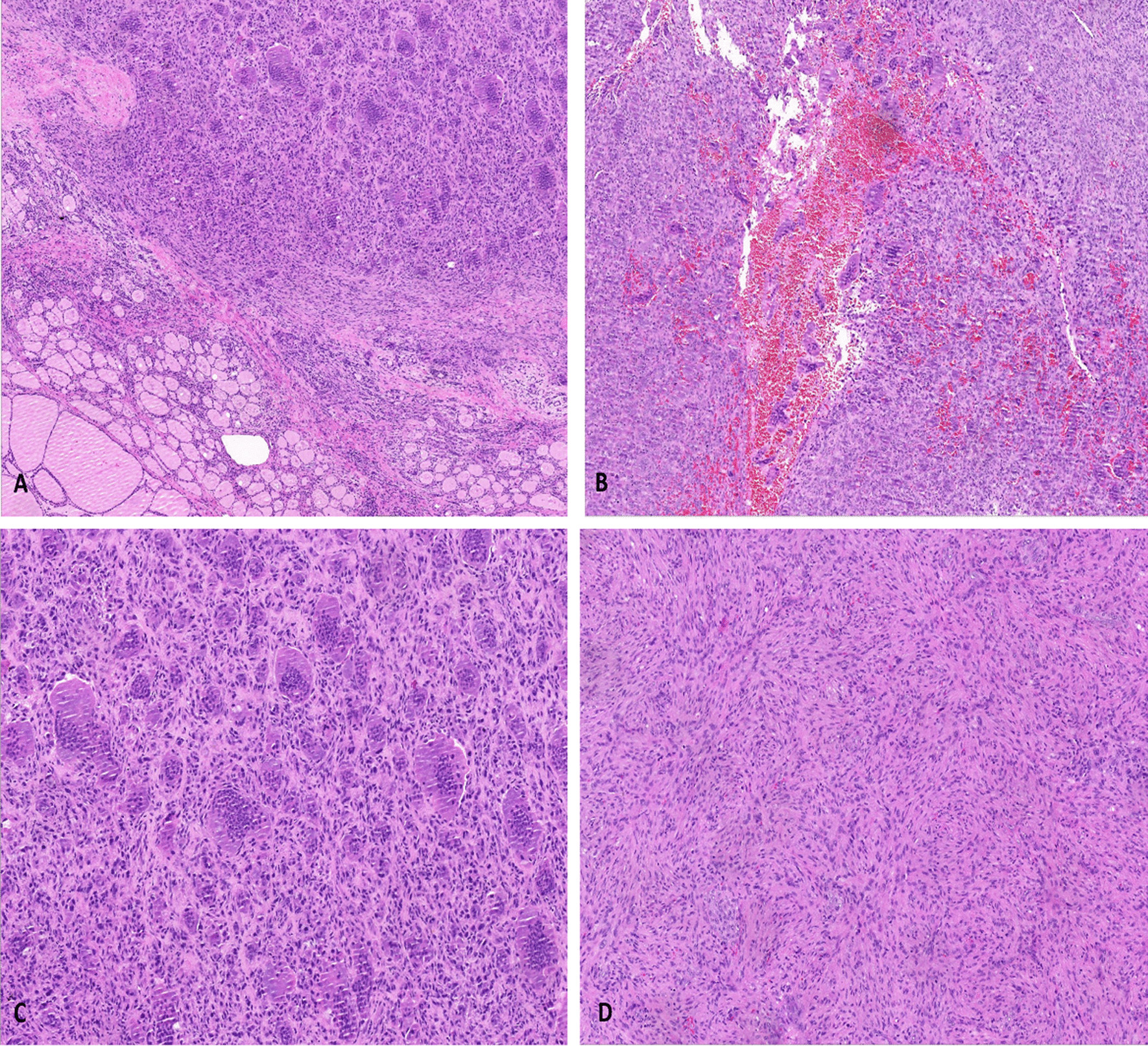
Histological features of thyroid giant cell tumor of soft tissue. **A** The tumor was composed of mononuclear stromal cells and a large number of multinucleated cells. **B** Hemorrhagic cystic formation is seen in the nodules, with marked interstitial fibrosis. **C** Osteoclast-like giant cells can be very large, with more than 50 nuclei. There are recognizable mitotic forms, but the cells exhibit minimal nuclear pleomorphism. **D** Mononuclear cells are histological and locally spindle shaped, with no atypia nor abnormal mitotic activity (hematoxylin and eosin, with original magnifications of 100×, 100×, 200×, and 200×, respectively)

Given the suspicion for thyroid carcinoma, she underwent left unilateral thyroidectomy. Intraoperatively, the tumor was located on the dorsal side of the left central pole of the thyroid gland, and clearly originated inside the thyroid. No obvious adhesion of the trachea and cricothyroid muscle was observed. Macroscopically, the mass measured approximately 35 mm × 20 mm × 18 mm in size and was obtained from the thyroid lobe. The cutting surface was solid and revealed a grayish brown, heterogeneous texture. Areas of necrosis and hemorrhage were not obvious. Histopathological examination with hematoxylin and eosin staining showed the tumor as multinodular and well circumscribed that in areas interlaced with the surrounding thyroid gland. The tumor was composed of mononuclear cells and multinucleated osteoclast-like giant cells. The nuclear morphology of mononuclear cells was similar to those of osteoclast multinucleated giant cells, and osteoclast giant cells were evenly distributed in the nodules (Fig. 2). Monocytes exhibited minimal pleomorphism in tumors, and mitosis was rare in our case, ranging from 1 to 2 per 10 high-power fields (HPFs). The osteoclastic cells were mainly found in oval-to-round monocytes. The number of nuclei in the multinucleated cells ranged from 3 to more than 50. In addition, these cells tended to cluster at the site of erythrocyte extravasation.

**Fig. 3:**
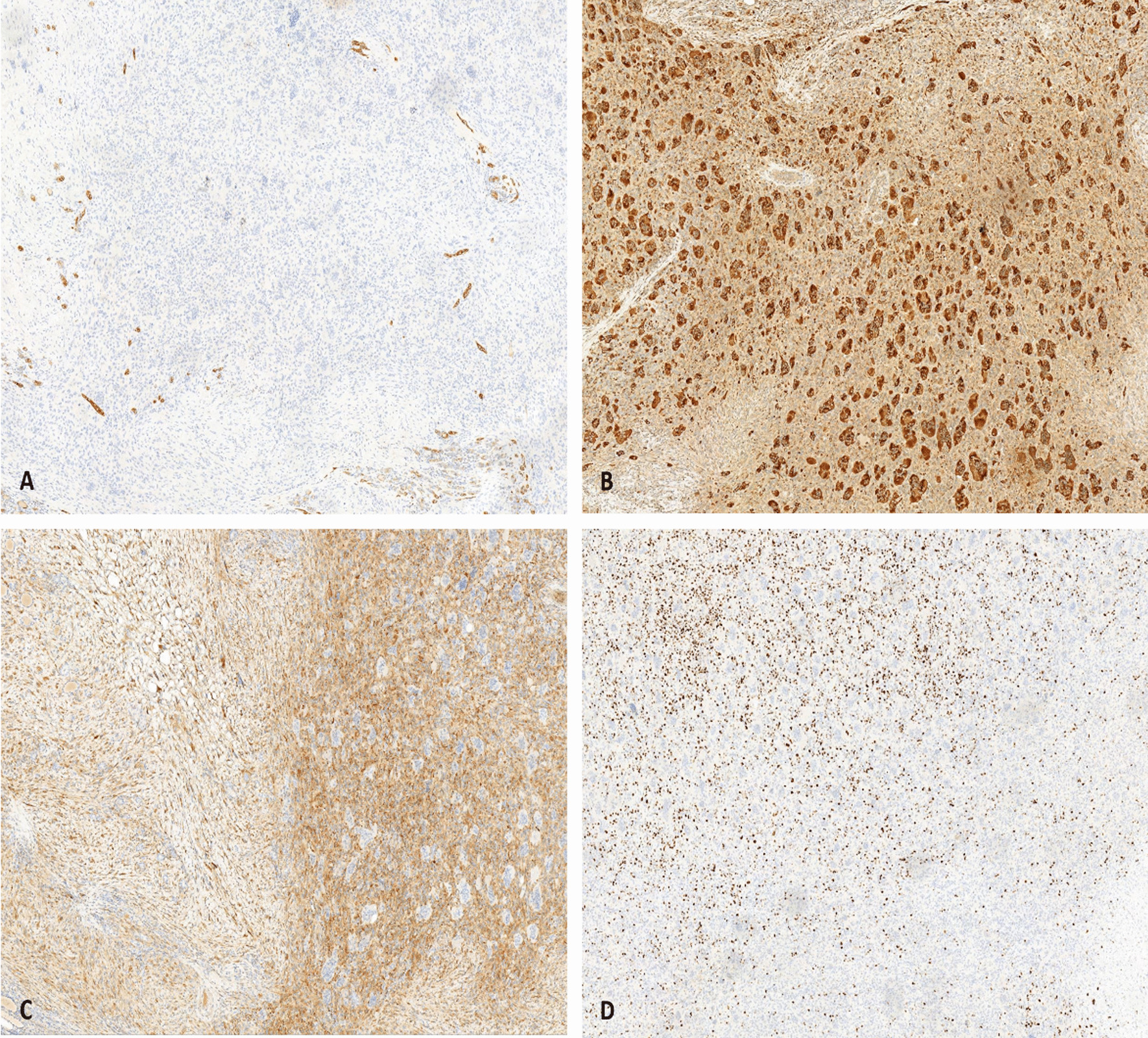
Immunohistochemical staining characteristics of thyroid giant cell tumor of soft tissue. **A** The tumor cells are negative for AE1/AE3 by immunohistochemical staining. **B** Immunostaining for CD68 is diffusely strong positive in multicellular cells. The mononuclear cells demonstrate reactivity for SYN **C** and the proliferation marker Ki-67 **D** mainly existed in the mononuclear cells (IHC staining, 100×)

On immunohistochemical analysis, these tumor cells were positive for CD68 and vimentin. The multicellular cells showed a higher expression of CD68 than that of monocytes (Fig. 3). The mononuclear cells were positive for synaptophysin (SYN), while negative for smooth muscle actin (SMA), desmin, and calcitonin. Cytokeratin AE1/AE3, thyroid transcription factor-1 (TTF-1), epithelial membrane antigen (EMA), S-100, and HMB45 were negative in the tumor cells. The Ki-67 labeling index ranged from 10% to 30%. Since there was no evidence of skeletal disease, we made a diagnosis of primary thyroid GCT-ST on the basis of the histological and immunohistochemical features.

The patient was admitted to Fudan University Shanghai Cancer Center with recurrence of left thyroid tumor 3 month later, and total thyroidectomy was performed. The tumor was diagnosed as primary GCT-ST of the thyroid and p-T1N0M0GX Stage IA. The postoperative process was satisfactory, and the patient was completely asymptomatic, without hypocalcemia or nerve damage. The patient did not receive adjuvant chemotherapy, and remained well without local recurrence or metastasis during 12 months of follow-up.

## Discussion

Giant cell tumor of soft tissue (GCT-ST) is a rare neoplasm commonly found in the superficial and deep soft tissue of the extremities [7–9]. GCT-ST affects both sexes equally and tends to occur in middle-aged adults. Since its morphologic characteristics and immunophenotype are similar to giant cell tumor of bone (GCTB), GCT-ST is regarded as its bony counterpart [10, 11]. Folpe *et al*. proposed the term “low-malignant potential giant cell tumor” for patients with biological behavior of frequent local recurrence and uncertain metastatic potential [4]. At present, these tumors are classified into low malignant potential GCT-ST and malignant GCT-ST according to the 5th edition of World Health Organization Classification of Soft Tissue and Bone [12].

It has been reported that GCT-ST occurs in various sites, such as salivary glands, pancreas, lung, and breast [13–16]. However, GCT-ST arising in the thyroid is particularly rare. In the English literature, two cases of giant cell tumor of bone involving the thyroid have been described: one in a 38-year-old male patient with a 35 mm diameter nodule located within the right thyroid lobe [17], and another in a 40-year-old male patient with a nodule in left thyroid lobe [18]. Both cases of thyroid GCTB were identified in the thyroid cartilage. Our patient also showed a nodular in the thyroid lobe, and underwent surgery to remove the thyroid along with the tumor. Clinical and radiological examinations revealed no evidence of bone involvement, and the tumor was found in the thyroid gland, not adjacent to the thyroid cartilage. Finally, the possibility of giant cell tumor of bone was ruled out in our case. Shahd *et al*. reviewed 12 cases of GCT-ST in the head and neck, and 5 were located in different neck triangles [19]. These tumors were all described as well circumscribed, and not related to the bone or cartilage.

Microscopically, GCT-ST consists of neoplastic mononuclear cells mixed with osteoclast-like multinucleated giant cells, both of cell types immersed in a vascularized matrix [11]. The mononuclear cells were short fusiform, with red stained cytoplasm and round-to-ovoid nuclei. Multinuclear giant cells, similar to osteoclasts, are larger in size, cytoplasmic eosinophilic, and had a central accumulation of dozens of pale or solid nuclei. Lau *et al*. proposed that the monocyte cell components fused to form multinuclear giant cells [10]. Mitotic activity was generally observed in GCT-ST, usually ranging from 1 to more than 30 per 10 HPFs. The tumor cells of malignant GCT-ST showed nuclear atypia, pleomorphism, and frequent abnormal mitotic activity. Immunohistochemical staining exhibited strong diffusely positive for CD68 within multinucleated osteoclast-like cells, whereas mononuclear cells were focal positive. All tumors were negative for a group of cytokeratin, epithelial membrane antigen (EMA), and leukocyte common antigen (LCA), suggesting mesenchymal lineage. Irene *et al*. analyzed the immunophenotype and molecular characteristics between GCT-ST and GCTB, and found that osteoclastogenesis was considered to be the result of fusion of preosteoclasts with CD14+ mononuclear cells [20]. However, GCT-ST might be genetically different from GCTB, and might be considered as two distinct entities in the study performed by Lee *et al*. [21].

The differential diagnosis of this disease includes benign lesion (subacute thyroiditis, true granulomatous such as tuberculosis or fungal infection, and sarcoidosis) and malignant lesions (papillary carcinoma, anaplastic carcinomas, and extraskeletal osteosarcoma). The two-cell pattern and immunohistochemical features of GCT-ST contribute to resolving this diagnostic difficulty. In our case, thyroid carcinoma was ruled out because of the absence of staining for epithelial markers. No significant nuclear atypia, pleomorphism, mitotic activity, and necrosis were observed, which ruled out the possibility of giant cell malignant fibrous histiocytoma. GCT-ST differs from plexiform fibrohistiocytic tumor (PFT) by lack of plexiform pattern of growth, larger nodules, metaplastic bone formation, and uniform distribution of multicellular osteoclast-like giant cell, which were absent in PFT. Previous studies have reported that small amounts of osteoid were found in some of GCT-ST, raising the differential diagnostic considerations for extra-skeletal osteosarcoma (ES-OGS). The giant cell-rich ES-OGS is a distinctly malformed tumor, with the morphologic features of conspicuous atypia and pleomorphism, as well as numerous bizarre, mitotic figures, which were not characteristic of GCT-ST. In ES-OGS, bone and osteoid formation is generated by the sarcomatoid, spindle cell stroma. In contrast, the osteoblast-lined, metaplastic bone trabeculae were seen in the cases of GCT-ST. Mana *et al*. described a case of extraskeletal giant cell tumor of the larynx, which definitely originated outside the laryngeal cartilage and was also diagnosed as a GCT-ST [22].

Since the majority of GCT-ST have a benign clinical course, the first-line treatment is complete surgical resection. There is no definitive evidence to support postoperative radiotherapy, but it should be considered in the case of incomplete surgical margin resection [5, 23]. Righi *et al*. found that the aggregation of osteoclast-like giant cells in GCTB and GCT-ST occurs through the RANKL-dependent mechanism, and drug inhibitors of osteoclast-like activity may control their absorption [20]. Several clinical studies and a retrospective case–control study have shown that denosumab (RANKL inhibitor) may reduce local recurrence after surgery [24], but there is no conclusive evidence for the use of denosumab in the recurrence of GCT-ST. In general, tumors lacking significant atypia or nuclear pleomorphism have benign behavior and rarely have local recurrences or lung metastasis. Guccion *et al*. found that tumors beneath a fascial plane were more aggressive [7]. On the contrary, the depth of involvement as having no significant effect on clinical course was reported by Folpe *et al*. The prognosis factors of GCT-ST are still unclear, and close follow-up is necessary due to the inability to predict local recurrence and metastasis.

In summary, primary thyroid GCT-ST is a rare tumor, composed of mononuclear and multinucleated cells. The clinical behavior of GCT-ST may vary widely, with some patients presenting with relatively indolent disease and others with rapidly distant metastasis. Familiarity with this rare disease and its characteristic histopathology features ensures establishing a correct diagnosis and distinguishing between other benign and malignant tumors. Clinicians and pathologists need to be aware of the rare diagnosis of GCT-ST in thyroid and should carefully individualize management and define its clinical prognosis.

## Conclusion

Giant cell tumor of soft tissue is a tumor with low malignant potential, which should be distinguished from other giant cell-rich soft tissue neoplasms. The significance of this case lies in the rarity of this entity, the challenge in preoperative diagnosis, and the differential diagnosis with other malignancies. More cases should be collected to improve the understanding of clinical characteristics, histopathology, pathogenesis, and relevant treatment.

## Data Availability

The datasets used and analyzed during the current study are available from the corresponding author upon request.

## Abbreviations

TSH: Thyroid stimulating hormone
FT3: Free triiodothyronine
FT4: Free thyroxine
Syn: Synaptophysin
SMA: Smooth muscle actin
TTF-1: Thyroid transcription factor-1
EMA: Epithelial membrane antigen
